# Editorial: Spontaneous coronary artery dissection: current state of diagnosis and treatment

**DOI:** 10.3389/fcvm.2024.1455983

**Published:** 2024-07-12

**Authors:** Svetlana Apostolovic, Srdjan Aleksandric, Branko Beleslin

**Affiliations:** ^1^ Cardiology Clinic, University Clinical Center of Nis, Nis, Serbia; ^2^ Medical Faculty, University of Nis, Nis, Serbia; ^3^ Cardiology Clinic, University Clinical Center of Serbia, Belgrade, Serbia; ^4^ Faculty of Medicine, University of Belgrade, Belgrade, Serbia

**Keywords:** spontaneous coronary artery dissection (SCAD), acute coronary syndrome (ACS), intravascular imaging, intravascular ultrasound (IVUS), optical coherence tomography (OCT), percutaneous coronary intervention (PCI), cutting balloon (CB)

**Editorial on the Research Topic** Spontaneous coronary artery dissection: current state of diagnosis and treatment

Spontaneous coronary artery dissection (SCAD) is relatively rare but potentially life-threatening condition characterized by the spontaneous separation of the layers of the coronary artery wall and the formation of intramural hematoma which compromises coronary blood flow (1, 2). Djokovic et al. discussed several mechanisms and theories to understand development of SCAD, including primarily structural weaknesses in the arterial wall with abnormalities in the connective tissue or smooth muscle cells predisposing spontaneous tearing or separation (“inside-out” or “outside in” hypothesis) (1, 2). Ultimately, the pathophysiology of SCAD likely involves a complex interplay of structural, hormonal, inflammatory, and genetic factors, highlighting the need for comprehensive research (3). Stanojević et al. discussed the most common predisposing factor like fibromuscular dysplasia, followed by inherited connective tissue disorders and systemic inflammatory diseases. Pregnancy and the use of sex hormones are common in younger females with SCAD. It was found that around 43% of acute coronary syndromes (ACS) cases among pregnant or postpartum women were caused by SCAD. It is also important to note that the presence of traditional risk factors for atherosclerosis does not exclude SCAD as a diagnosis in young patients with ACS.

Invasive coronary angiography remains the most important diagnostic tool in suspected SCAD, and Kovacevic et al. discussed the angiographic presentation of SCAD. According to Yip-Saw classification (4), there are three typical angiographic patterns of SCAD, but several potential pitfalls and essential differential diagnoses should be considered. Type 1 SCAD is characterized by a pathognomonic angiographic appearance and a recognizable radiolucent flap, usually affecting the proximal segments of coronary arteries. Type 2 SCAD is the most common type, presents as a smooth diffuse stenosis either with lumen restoration in the distal segment (Type 2a) or stenosis extending till the end of the artery (Type 2b). In addition to atherosclerosis, the most common mimic of SCAD type 2 is coronary vasospasm (focal or diffuse), which can be distinguished with intracoronary nitroglycerine injections. Type 3 SCAD is characterized by focal stenosis and underlying hematoma resembling a ruptured atherosclerotic plaque and is frequently missed by coronary angiography alone. Therefore, to distinguish features that mimic SCAD, high-resolution intracoronary imaging techniques such as intravascular ultrasound (IVUS) or optical coherence tomography (OCT), may be beneficial. Recently, additional type 4 SCAD has been proposed to describe total vessel occlusion, usually of a distal coronary artery (5). It is particularly challenging to diagnose, and is often misinterpreted as an atherosclerotic occlusion, thus being treated systematically by percutaneous coronary intervention (PCI). As type 4 SCAD often coexists with other SCAD types or occurs as a consequence of their progression, an intramural hematoma near the occlusion could be identified with intravascular imaging techniques. Krljanac et al. described the role of multimodality imaging, especially echocardiography and cardiac magnetic imaging (CMR), in the evaluation and follow-up of SCAD patients presenting with ST-elevation myocardial infarction (STEMI) (6). Previous studies showed that the majority of these patients have a mild myocardial infarctions and preserved or slightly impaired left ventricle (LV) systolic function (7). Therefore, the improvement in LV systolic function during follow-up is greater than that seen in patients with type 1 STEMI. These differences may be related to a higher prevalence of TIMI 3 flow at coronary angiography and an overall smaller ischemic burden in STEMI patients caused by SCAD than in those caused by erosion/rupture of the atherosclerotic plaque and subsequent thrombosis. However, there is no assurance that this applies to more complex types of SCAD, such as total vessel occlusion and multisegmental or multivessel engagement.

**Figure 1:**
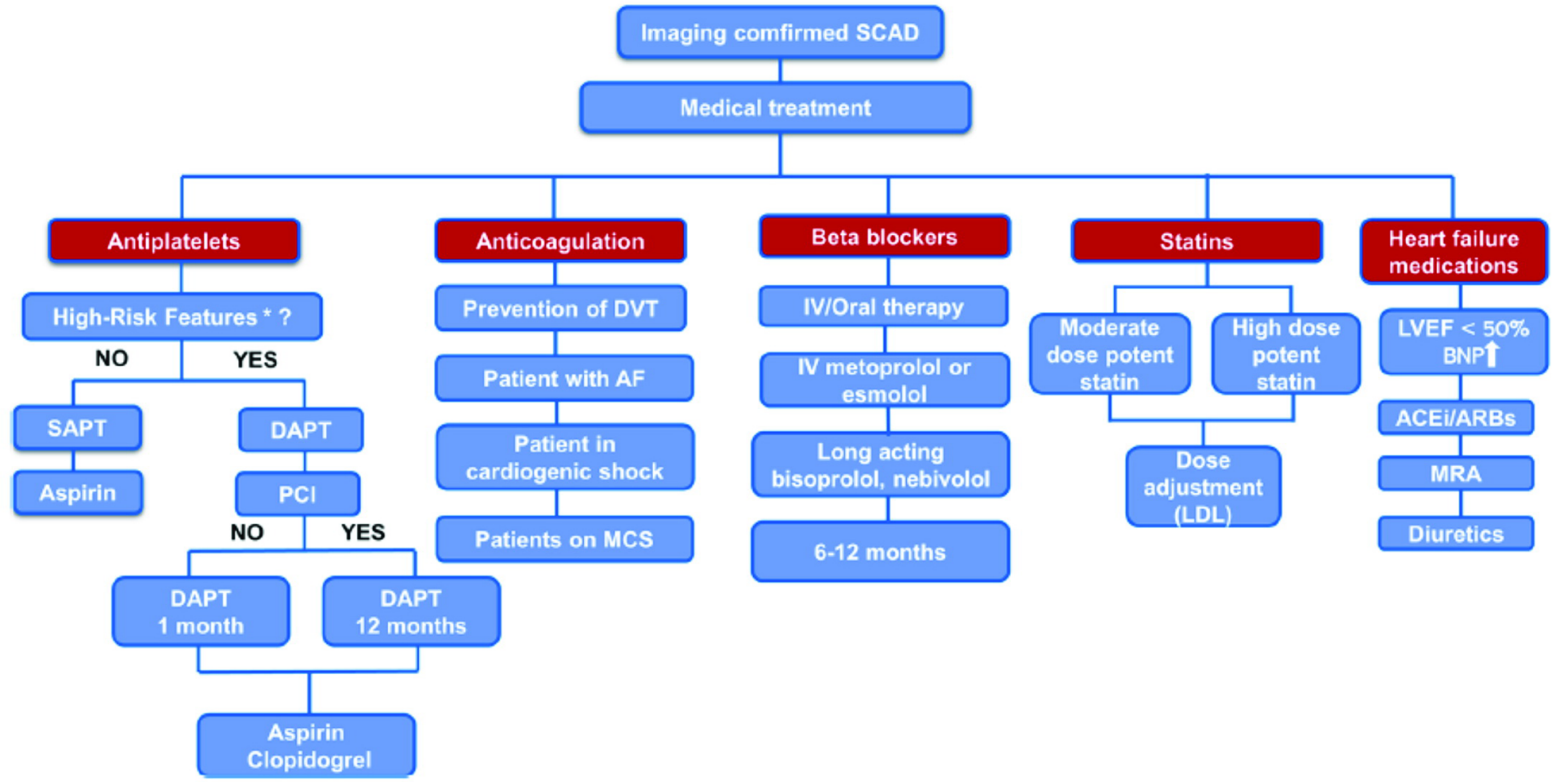
Proposed algorithm for medical treatment of imaging confirmed SCAD. *“high-risk” features - concomitant atherosclerosis, large thrombus burden, critical stenosis that was left untreated, and significant flow impairment in the affected coronary artery. AF, atrial fibrillation; ACEi, angiotensin-converting enzyme inhibitors; ARBs, angiotensin receptor blockers; BNP, brain natriuretic peptide; DAPT, dual antiplatelet therapy; DVT, deep vein thrombosis; IV, intravenous; LVEF, left ventricle ejection fraction; MCS, mechanical circulatory support; MRA, mineralocorticoid receptor antagonist; PCI, percutaneous coronary intervention; SAPT, single antiplatelet therapy.

Mehmedbegovic et al. pointed out the importance of intravascular imaging techniques (IVUS and OCT) in the differential diagnosis between SCAD and other coronary lesions such as atherosclerotic plaque with or without intracoronary thrombus or myocardial bridging. The main disadvantage of invasive coronary angiography is that it is basically just a “luminography” that provides little information regarding artery wall integrity. Quite the opposite, IVUS and OCT would provide detailed phenomena typical of SCAD-like lesions such as the existence of an intimal flap, the presence and extent of intramural hematoma and/or thrombus, and the absence of atherosclerotic changes in the arterial wall. Intravascular imaging should therefore only be used if angiographic findings are unclear in large arteries (especially in SCAD types 3 and 4) and/or if further PCI is required (8–11). Current treatment strategies for SCAD patients were explained in detail in a comprehensive review by Ilic et al. (Figure 1). While no randomized clinical trials have been conducted on medical treatment for SCAD, treatment strategies generally emphasize a conservative approach since spontaneous healing of SCAD usually occurs in the first 30 days after the event (12, 13). Percutaneous coronary intervention is recommended for patients with ongoing ischemia and/or hemodynamic instability due to its high complication rates and low angiographic success rates (12, 14). However, the multicentre international “DIssezioni Spontanee COronariche (DISCO)” registry, which included 314 SCAD-patients, found that dual antiplatelet therapy (DAPT) was associated with a 2.6-fold higher risk for major adverse cardiovascular events (MACE) compared to single antiplatelet therapy (SAPT) with mainly aspirin at 1-year follow-up (15). These findings implicate that DAPT could be harmful in conservatively managed SCAD patients, especially those with intramural haematoma due to intramural bleeding aggravation, haematoma and dissection propagation and subsequent arterial lumen compression (15). Therefore, there is consensus that DAPT should be prescribed in SCAD patients, consisting of aspirin and clopidogrel, and should be limited to the first 30 days following hospital admission, except for those with stent implantation who should be treated in accordance with the current guidelines for ACS (1, 14). It is recommended to continue taking aspirin monotherapy after 1 month, but the duration of this therapy remains unknown. The current recommendations also support the use of beta-blockers as a first-line therapy for at least 1 year after the event since their use was associated with a significantly lower risk of SCAD recurrence (16). Other medications such as angiotensin-converting enzyme (ACE) inhibitors, angiotensin-receptor blockers (ARB), mineralocorticoid antagonists, loop diuretics and statins are recommended for patients with concomitant risk factors for atherosclerosis and coronary artery disease and/or heart failure with reduced or mild ejection fraction (EF<50%), in accordance with the current guidelines.

A percutaneous angioplasty using a cutting balloon as a novel interventional strategy for the treatment of SCAD was described by Maricic et al. This technique entails positioning of a cutting balloon inside the true lumen to cause controlled micro-incisions within the affected vessel, causing intimal fenestration and hematoma draining (1, 17). Consequently, the true arterial lumen is decompressed, and coronary blood flow is restored. According to current data, by using a smaller cutting balloon than the reference vessel diameter, the risk of vessel injury can be minimized, and the procedure can be more effective. The most often procedure complication is distal propagation of the subintimal hematoma with dissection extension, while coronary perforation and acute vessel closure are very rare. If such a situation arises, stenting may be the only option to stabilize the dissected coronary artery and provide additional support. Further research is needed to determine the long-term clinical implications and compare the efficacy and safety of cutting balloon angioplasty with other treatment options for SCAD.

A systematic review by Petrovic et al., which included 13 observational studies, examined clinical outcomes in 1,801 patients with SCAD treated conservatively (65%) or invasively (PCI 33%; coronary artery bypass grafting 1.3%). Percutaneous coronary intervention was associated with a higher rate of periprocedural complications, mostly hematoma extension and/or iatrogenic dissection, which frequently required the implantation of at least three stents with residual areas of dissection. The overall reported in-hospital and follow-up mortality rates were 1.2% and 1.3%, respectively. According to these results, conservative treatment is the preferred treatment option for patients with SCAD. A review by Apostolovic et al. focused on female patients in generative period (16–55 of age) with ACS caused by SCAD and compared clinical characteristics and outcomes between non-pregnant women with SCAD and pregnant women with SCAD. Compared to non-pregnant women, pregnant women have a greater chance of having SCAD in the left main and/or the left anterior descending artery (LAD); are more likely to have STEMI; and are more likely to undergo PCI. However, there were no differences regarding mortality rates or recurrent coronary dissection between these two study groups. Future research efforts with developing specialized SCAD registries will contribute to a better understanding of this condition and its outcomes.
